# Tetrachromacy and advertising: a new way of visual perception in marketing

**DOI:** 10.22336/rjo.2024.63

**Published:** 2024

**Authors:** Consuela-Mădălina Gheorghe

As mentioned in the literature, we have witnessed a growth in interest in human tetrachromacy in recent years. This condition is often called a “superpower” of individuals, who have been proved to be mostly women. Tetrachromacy can be regarded as both a condition if we are discussing the ailment per se and a “superpower” if we are discussing the signals in the skin tone of conspecifics that are linked to health or disease (Jordan G, Mollon J. Tetrachromacy: the mysterious case of extra-ordinary color vision. Current Opinion in Behavioral Sciences. 2019; 30:130-134. https://doi.org/10.1016/j.cobeha.2019.08.002), the interpretation of human emotion states or choosing the colors and shades of color in online and offline advertisements. However, doctors nowadays use color stains in cell histology and color codes on medical instruments to detect diseases, but the ones who are color deficient might miss symptoms of patients because of their inability.

Tetrachromacy comes from the Greek words tetra, which means “four”, and chroma, which means “color”. It refers to the state in which the eye has four distinct channels for communicating color information or four different kinds of cone cells. Tetrachromats are organisms that possess tetrachromacy.

Tetrachromatic creatures have a four-dimensional sensory color space, which means that combinations of at least four primary colors are needed to match the sensory effect of randomly selected light spectra within their visual spectrum.

Numerous fish species, reptiles, and bird species have been shown to exhibit tetrachromacy (Goldsmith TH. What Birds See. Scientific American. July 2006; 69-75; Bowmaker JK. Evolution of vertebrate visual pigments. Vision Research. September 2008; 48(20):2022-2041. doi: 10.1016/j.visres.2008.03.025. PMID: 18590925).

Tetrachromacy’s physiology is typically explained by the presence of four different types of higher-intensity light receptors with varying spectral sensitivity in the retina of the organism (referred to as cone cells in vertebrates as opposed to rod cells, which are lower-intensity light receptors). This implies that the creature might be able to perceive wavelengths outside the range of the average person's vision and could discriminate between colors that appear identical to a regular human. Tetrachromatic color vision may give some species a physiological edge over competitors (Backhaus W, Kliegl R, Werner JS. Color vision: perspectives from different disciplines. De Gruyter. 1998; 163-182).

While talking about tetrachromacy in humans, it should be noted that Old World monkeys and apes, including humans, typically only have three types of cone cells, making them trichromats. However, a tiny portion of the population is thought to have human tetrachromacy. According to the diagram, trichromats have three different kinds of cone cells, each sensitive to a different spectrum region. However, it has been suggested that at least one woman is tetrachromatic (Jordan G, Deeb SS, Bosten JM, Mollon JD. The dimensionality of color vision in carriers of anomalous trichromacy. Journal of Vision. July 2010; 10(12):12. doi: 10.1167/10.8.12. PMID: 20884587). More specifically, she exhibited three-dimensional (M, L', and L components) color discrimination at wavelengths 546-670 nm (to which the fourth type, S, is unresponsive) and an additional cone type L′, which is midway between M and L in its responsivity.

Recent research has shown that the tetrachromatic women excel in fields of design, visual arts and advertising, thus giving them the “power” to choose the perfect shade or color when designing print and online advertisements, because in industries like advertising, visual appeal and consumer engagement are very important.

Regarding the implications for advertising, it should be restated that advertising has always relied on color to influence consumer behavior, trigger emotions and define brand identity. The best examples are the deep red that Coca-Cola uses for its main product or the calming blue that Facebook uses for its online page. For these examples and for many other, the conclusion is that colors have psychological associations that the marketers exploit to establish a connection with their audiences. Tetrachromacy offers a new frontier to be explored by advertisers who can design campaigns and products that are more appealing to those with this enhanced color perception.

Tetrachromats may react more strongly to advertisements that use these subdued colors since they can see a wider variety of colors. Marketers may, for instance, experiment with color schemes that include tones that are only visible to tetrachromats, giving ads and goods a more upscale and distinctive appearance. Finer gradients or invisible UV colors could be used to reach this audience in a way that traditional advertising is unable to. Brands might serve a very specialized and visually savvy clientele by embracing the diversity of color perception.

In advertising, the psychology of color is a well-established discipline. From the warmth and vitality of red to the reliability and serenity of blue, different colors evoke distinct feelings and memories. Tetrachromats may be more sensitive to these psychological cues because of their improved capacity to perceive subtler color changes. Marketers might experiment with more nuanced color selections, employing extra shades that elicit distinct feelings in this target market. For example, a subtle shift from violet to purple that is only apparent to tetrachromats may have a soothing, nearly meditative effect that could appeal to a niche demographic that is interested in relaxation or wellbeing.

Color can be a very effective strategy for developing unique brand identities as companies look to stand out in a competitive market. A new kind of brand experience that capitalizes on visual richness and individuality is possible with tetrachromacy. Companies might create logos, ads, and merchandise with color schemes that are only noticeable to people who have tetrachromatic vision, giving the impression that interaction with the brand is more specialized and exclusive. This might lead to a brand-new luxury marketing strategy in which color is a crucial difference.

The possibilities offered by tetrachromacy may cause a major change in the way ads are created. Fundamentally, visual communication involves using shapes, colors, and images to convey a message. Advertisements could include subtle color and pattern changes that are currently unavailable to the general population for tetrachromats, who see an extended visual range. Ads may feel more lively, energetic, and profound because to these delicate hue changes, providing viewers with a deeper visual storytelling experience that speaks to them more intimately.

Tetrachromacy in advertising has amazing promise, but there are drawbacks and moral dilemmas. First, since the condition is uncommon, focusing just on tetrachromats may alienate the great majority of consumers who do not have this enhanced capacity. Advertisers would therefore have to carefully weigh the advantages of interacting with this niche but distinct audience against the more general requirements of their target market. Furthermore, using invisible colors can be interpreted as exclusive or manipulative, which would call into question the impartiality and accessibility of advertising tactics. When using tetrachromacy in advertising, it is important to make sure that the ads stay inclusive and do not unintentionally leave out or take advantage of certain groups.

The advertising business could undergo a big transformation due to tetrachromacy, which is the capacity to sense a greater variety of colors than most individuals. Advertisers might develop more sophisticated, emotionally impactful, and exclusive advertisements that appeal to a certain demographic by utilizing the distinctive visual experiences of tetrachromats. The use of this sophisticated visual perception must be handled carefully, though, to improve the customer experience without offending or deceiving the public. The potential for utilizing novel color perception techniques in the advertising industry will grow along with our comprehension of vision.

**Assist. Prof. Consuela-Mădălina Gheorghe, PhD, Philologist, Certified translator**

